# Annotations

**Published:** 1896-11-28

**Authors:** 


### ANNOTATIONS.

### Local Government.

The medical profession, partly by virtue of its intimate personal relations therewith, but more because of its eager interest in all questions which have to do with the health and prosperity of the community, is keenly interested in all the details which go to make up what is known in the mass as "Local Government." The report just issued for 1896 contains some very encouraging reading. The whole country, as some of our readers may be aware, is now divided into fourteen administered districts; and each district is supervised by a "general inspector." There is here a definite system, capable of focussing work and results; and it is the summarised reports of the inspectors which enable us to see what is being done, and to note with satisfaction how much of general well-being there has been among the masses of the people during the past year. One conspicuous fact well illustrates their satisfactory condition as compared with their state, not merely in recent years, but in the earlier part of the century; and that is the steady diminution in pauperism, and the relative health and happiness of that large class who are always on the verge of pauperism. The year 1895, as may be remembered, opened with a tremendously severe frost, which continued more or less for ten weeks. Such a period of prolonged and bitter cold tested the resources of the less prosperous portion of the working classes to the utmost; and in times not very remote from our own would undoubtedly have produced paupers in numbers almost impossible to be dealt with. But in spite of this and many other conditions, tending directly to the encouragement of pauperism, the actual increase in the number of paupers was merely fractional. All this speaks well for the utility of a thorough and detailed system of local government.

### The Diagnosis of Typhoid Fever.

London compares but badly with New York in the activity of its sanitary administration. While we yet hesitate, and still remain without any central laboratory for the bacteriological diagnosis of diphtheria, and just at the moment when the only attempt at any organisation of the sort which we possess, viz., the arrangement which has been in operation for the last two years between the Asylums Board and the Laboratory of the Royal Colleges, is going to be knocked on the head, we find the authorities in New York adding to their arrangements for the bacteriological diagnosis of diphtheria and tuberculosis the important work of diagnosing typhoid fever. On November 7th Dr. Hermann Biggs, the director of the Bacteriological Department, drew the attention of the Health Department to the results obtained by Pfeiffer and Widal in this direction, and urged that facilities should be placed at the command of all physicians in the city for the examination of the blood of patients suffering or suspected to be suffering from typhoid fever by the method recommended. The difficulties in the way of diagnosis in obscure cases of typhoid fever are often extremely great, and it is well understood that it is largely by the slight cases which although ailing are able to walk about, that the infection is distributed. From the public point of view the importance of a definite test is extreme. This was at once recognised in New York, where Dr. Biggs' recommendation was immediately acted on. Here we are more lethargic. We take our troubles as they come, but do little to prevent them; and while we are spending immense sums every year in the treatment of diphtheria we have not yet screwed up our courage to spend the few hundreds necessary for the establishment of a municipal laboratory by which to decide whether or not patients thought to have diphtheria have got the disease at all.

### London Water and the Experts.

It is a pity that the question of the purity of the London water should drift, as it tends to do, into a mere personal matter as to the methods and accuracy of the various specialists who have been engaged in its investigation. Perhaps it is to be regretted that the investigators employed by the County Council should have waved their tomahawks so triumphantly over those employed by the water companies and the Government. On the other hand, it is equally to be regretted that the latter, while attacking the bacteriological methods of their assailants, should have ignored the fact that the complaint of bad filtering did not depend on these alone, but on the presence of matters much larger than bacteria. The bacteriological question is an interesting one, and one that ought to be threshed out; but the charge made against the London water is not that it is merely bacteriologically impure, but that it is at times so badly filtered as to contain wool and cotton fibres, the larger infusoria, anguillulæ, and "briskly moving" animalculi—creatures so large that, where they can pass, bacteria in almost unlimited quantity could get through. The teaching of these reports is quite as much to the effect that by care the London water can be made good as it is to the effect that at present it is often bad. The quarrel between the experts is a matter which they must fight out between themselves. But we may point out that the greater our confidence in the accuracy of Dr. Frankland's bacteriological examination, and the more our faith in the reports published from time to time by the chemists employed by the water companies as to the purity of the samples examined by them, the stronger does the accusation against the companies become of having mixed the waters, either deliberately or from want of care. If the water was pure, clean, and free from sediment, as it flowed from the filters, and if when it was drawn from the pipes it contained diatoms, and little worms, and "fragments of matter in a state of decomposition," all of them things found plentifully in the river water, and far too large to have passed through a reasonably fine filter, it becomes difficult to avoid the conclusion that by some mismanagement imperfectly filtered river water has been allowed to mix with that which has gone through the filters. It is in this charge of carelessness against the companies, and in the hope thus held out, that the London water, even from its present source, could be much improved if the companies would but take the trouble and go to the necessary expense, that the interest of these reports lies to Londoners at large—not in the imputations of imperfect work cast by one expert upon another.

